# 33 Standardizing an Early Ambulation Protocol for Lower Extremity Grafts

**DOI:** 10.1093/jbcr/irae036.033

**Published:** 2024-04-17

**Authors:** Audrey M O'Neil, Cassandra Rush, Laura Griffard, Brett Hartman

**Affiliations:** Richard M. Fairbanks Burn Center, Indianapolis, IN; Eskenazi Health, Indianapolis, IN; Richard M. Fairbanks Burn Center, Indianapolis, IN; Eskenazi Health, Indianapolis, IN; Richard M. Fairbanks Burn Center, Indianapolis, IN; Eskenazi Health, Indianapolis, IN; Richard M. Fairbanks Burn Center, Indianapolis, IN; Eskenazi Health, Indianapolis, IN

## Abstract

**Introduction:**

Early post-op ambulation benefits burn survivors by allowing quicker return to independent ambulation, limiting joint stiffness associated with immobilization, and preventing complications from bedrest. Burn Practice Guidelines for early ambulation were published in 2012, however significant variability in practice continues with burn centers reporting initial ambulation occurring between 0-14 days post-op, averaging on day 3 when the lower leg and foot is involved. Additionally, immobilization and weight bearing restrictions are recommended if the autograft is placed over a joint.

**Methods:**

A retrospective review was completed of this 15 bed, adult verified burn center, from September 2020-August 2023 to identify patients who underwent split thickness skin graft (STSG) placement and/or Autologous Skin Cell Suspension (ASCS) application to their lower extremities (LE). Patients who were unable to ambulate at baseline or died during admission were excluded. Medical records were then reviewed for injury related factors, surgical interventions, post-op protocols, mobility initiation, discharge location, and wound healing using photo documentation.

**Results:**

During the 3-year period, 149 patients with LE injury met the criteria for retrospective review. LE involvement ranged from 1-28% TBSA (Avg 6.9%). Initial homograft or dermal substitute placement was required for 56.3% (n=84) with autograft placement occurring on average post-burn day 8.5. Autograft placement included 68% Meshed (1:1-3:1) STSG (n=101), 16% Meshed STSG with ASCS (n=24), 15% ASCS (n=22), and 1% Sheet STSG (n=2). Grafts crossed joints on 119 patients including the knee (n=58), ankle (n=57), and foot (n=41). Initial ambulation occurred on average POD 1.05 using either Coban (n=68) or an Unna Boot (n=47) for compression support. Initial gait distance ranged from 2 to 1,500 ft (avg 125.4 ft). LE joint immobilization was limited to 18 patients due to post-surgical precautions and 8 patients due to active nerve blocks. No graft loss was associated with post-op ambulation. Twelve patients experienced minor graft loss, attributed to injury or surgical factors, infection, and patient comorbidities, which healed conservatively.

**Conclusions:**

Post-op ambulation in burn patients can occur earlier than documented in previous burn research without immobilization or weight bearing restrictions. LE graft loss was not found to be associated with early post-op ambulation. Current recommendations include compression application to the lower extremity for vascular support and initiation of scar management. Immobilization can be used in cases of tendon exposure or joint compromise.

**Applicability of Research to Practice:**

Variability in practice among burn centers regarding mobility protocols prevents adoption of early ambulation as the new standard of care. Combining rehabilitation interventions with use of compression to support STSG can allow unrestricted early ambulation.

